# Analysis of modified second-grade fluids in thin film configurations employing the asymptotic homotopy perturbation method

**DOI:** 10.1038/s41598-026-39180-5

**Published:** 2026-03-06

**Authors:** M. Mossa Al-sawalha, Musaad S. Aldhabani, Ahmed M. Zidan, Abdulkafi Mohammed Saeed, Ahmed Shafee, Noorullah Noori

**Affiliations:** 1 Department of Mathematics, College of Science, University of Ha’il, 2440 Ha’il, Saudi Arabia; 2 Department of Mathematics, Faculty of Science, University of Tabuk, P.O.Box741, 71491 Tabuk, Saudi Arabia; 3 Department of Mathematics, College of Science, King Khalid University, P.O. Box: 9004, 61413 Abha, Saudi Arabia; 4 Department of Mathematics, College of Science, Qassim University, 51452 Buraydah, Saudi Arabia; 5 Laboratory Technology Department, College of Technological Studies, PAAET, 70654 Shuwaikh, Kuwait; 6 Faculty of Mathematics, Kabul University, Kabul, Afghanistan

**Keywords:** AHPM, Thin film flow, Lifting, Drainage, Engineering, Mathematics and computing, Physics

## Abstract

This article investigates the steady, incompressible and non-isothermal thin film flow of a modified second grade fluid under both conditions that is, lifting and drainage. The governing highly non-linear ordinary differential equations with appropriate boundary conditions have been solved and achieved approximate solutions employing the Asymptotic Homotopy Perturbation Method (AHPM). The velocity and temperature distributions have been obtained, moreover average velocity, shear stress and volume flow rate at the belt surface are presented graphically for both lifting and drainage configurations. Here it is worth mentioning that the flow of thin films are significantly influenced by $m$ (flow behavior index of modified second grade fluid), Brinkman number and Stokes number. The outcomes of this work are relevant to several industrial and engineering applications, comprising coating and drying technologies, polymer extrusion and processing, lubrication systems, chemical and food processing, and thermal control in thin-film transport processes. Additionally, graphical and numerical analyses have been provided to explain the impact of various parameters on the fluid behavior.

## Introduction

Thin-film flows of non-Newtonian fluids are essential in numerous industrial applications, extending from lubrication processes and coating to cooling system and polymer extrusion. These flows developed exclusively complex when comprising fluids that show elasticity, shear-thinning or shear-thickening behavior, and when heat transfer effects are significant. Latest studies on thin-film and non-Newtonian fluid heat transfer have concentrated on similarity transformations that shrink the governing partial differential equations to nonlinear ordinary differential equations, which persist exciting owing to strong hydrodynamic–thermal coupling. For instance, unsteady thin-film flow and heat transfer of power-law hybrid Nano-fluids over stretching surfaces have been explored numerically, emphasizing the possessions of slip and heat transfer parameters on flow performance^1^. Such studies extend the understanding of how the non-Newtonian flow behavior and thermal transport relate in thin films exposed to stretching and thermal gradients, giving valuable representations for advanced engineering uses^2^.

Researchers in^3^ investigated entropy generation in bio-convective Walters’ B Nano-fluid flow employing the Cattaneo–Christov model, highlighting thermal relaxation effects. Scientists in^4^ studied radiative micro-polar fluid flow over a curved stretching sheet, emphasizing the influence of viscous dissipation and micro-rotation. Authors in^5,6^ examined MHD hybrid Nano-fluid flows and Nano-particle shape effects in porous media with slip, signifying enhanced heat transfer and flow control. Together, these investigations support the theoretical and computational understanding of non-Newtonian and Nano-fluid flow schemes. Numerous studies show the efficiency of ANN models in forecasting thermo-viscous and transient flow performance between parallel walls and porous domains^7–9^. Authors in^10,11^ investigated the Unsteady flows around oscillating spheres using analytical and numerical methods, providing important understandings into thermo-viscous effects. Researchers in^12–17^ further address heat generation, non-Newtonian, MHD and Nano-fluid flows with thermal radiation, and chemical reactions along with uses in bio-convective transport and phase change heat energy storage.

To discourse this, semi-analytical methods, for example homotopy-based methods, have attained extensive recognition. The Homotopy Perturbation Method (HPM) was presented to well tackle non-linear differential equations by constructing a homotopy with an embedding parameter. Its convergence characteristics and comparatively simple application have made it prevalent in fluid dynamics and heat transfer problems^18,19^. HPM has earlier been employed to examine thin layers of non-Newtonian fluids. For instance: Researchers in^20^ employed HPM to examine thin-film flow of a third-grade fluid down an inclined plane, getting analytical solutions. Researchers in^21^ extended HPM to thin-film flow of a fourth-grade fluid around a vertical cylinder, demonstrating greater performance over traditional perturbation techniques. Though, there leftovers a notable lack of applications including modified second grade fluids in non-isothermal thin-film conditions and without depending exclusively on HPM.

In this investigation we employs the Asymptotic Homotopy Perturbation Method (AHPM)) AHPM was selected owing to its structured hierarchical outline, reliability-checking competence, and extensive approval in the literature, making it appropriate for the present investigation. It is an advanced variant of HPM that presents clear asymptotic control and convergence acceleration to explore the highly nonlinear ODEs governing the non-isothermal thin-film flow of a modified second-grade fluid in both lifting and drainage cases:

Main goals of our procedure contain:

1. Building a strong homotopy embedding with an asymptotic parameter to confirm convergence across parameter regimes.
2. Developing semi-analytical series solutions for velocity distribution and temperature profiles, and graphical presentation of volumetric flow rate, and shear stress.
3. Investigating the effect of dimensionless parameters for instance the Stokes number, the fluid’s flow behavior index and the Brinkman number
4. Illustrating results numerically as well as graphically to explain that how these parameters modify flow behavior in both cases.

## Research gap

A vigilant review of the existing literature tells that most researches on thin film flows emphasis on Newtonian or simple non-Newtonian fluids under isothermal conditions and often study either lifting or drainage structures separately. The non-isothermal thin film flow of a modified second-grade fluid, accounting simultaneously for both lifting and drainage effects plus influenced by parameters for example the flow behavior index, Brinkman number, and Stokes number, has acknowledged minute attention. The present work addresses this gap by providing an analytical dealing employing the Asymptotic Homotopy Perturbation Method.

## Basic idea of AHPM

We chose the following differential equation:

1 $$L(v(x)) + g(x) + K(v(x)) = 0.$$

So as to describe the basic notion of AHPM, where $$g(x)$$ is known function, $$v(x)$$ is the unknown function, $$L$$ is linear operator and $$K$$ is nonlinear operator. Now by homotopy $$\phi (x,p):\Omega \times [0,1] \to R[0,1]$$$$\to R$$^22^ $$\mathrel\backepsilon$$

2 $$L(\phi (x,p)) + g(x) - p[K(\phi (x,p))] = 0,$$

where the embedding parameter is $p \in [0,1]$. Now by OHAM proposed by Marinca^23^ as

3 $$\left( {1 - p} \right)\left[ {L\left( {\phi (x,p)} \right) + g(x)} \right] - W(p)\left[ {L\left( {\phi \left( {x,p} \right)} \right) + g(x) + K\left( {\phi (x,p)} \right)} \right] = 0.$$

Clearly as $p = 0$ along with $p = 1$ we have

$$\phi \left( {v(0)} \right) = v_{0} (x),\;\left( {\phi v(1)} \right) = v.$$

Chose $\phi \left( {v(p)} \right)$ is in the form

4 $$\phi \left( {v(p)} \right) = v_{0} + \sum\limits_{l = 1}^{\infty } {v_{l} p^{l} } ,$$

$K(\phi (x,p))$ that is, the non-linear part

5 $$K(\phi (x,p)) = G_{1} K_{0} + \sum\limits_{l = 1}^{\infty } {\left( {\sum\limits_{n = 0}^{l} {G_{l + 1 - n} K_{n} } } \right)} \,p^{l} ,\,\,G_{1} + G_{2} + G_{3} + ... = - 1.$$

where

6 $$G_{l} = G_{l} (x,h_{l} ),\,\,l = 1,2,3,....$$

Putting (4) and (5) in (2) and equating same exponents of $p,$ as

$$p^{0} :L(v_{0} ) + g = 0,$$

$$p^{1} :L(v_{1} ) = G_{1} K_{0} ,$$

$$p^{2} :L(v_{2} ) = G_{2} K_{0} + G_{1} K_{1} ,$$

$$p^{3} :L(v_{3} ) = G_{3} K_{0} + G_{2} K_{1} + G_{1} K_{2} ,$$

In general

$$p^{l} :L(v_{l} ) = \sum\limits_{j = 0}^{l - 1} {G_{l - j} K_{j} } .$$

Equation (2) at $p = 1$ converges to Eq. (1), specifically the exact solution

7 $$v(x,f_{i} ) = v_{0} (x) + \sum\limits_{l = 1}^{\infty } {v_{l} (x,f_{m} ),} \quad m = 1,2,3 \ldots$$

After using Eq. (7) in Eq. (1) the residual as

8 $$R\left( {x,f_{l} } \right) = L\left( {v\left( {x,f_{l} } \right)} \right) + g\left( x \right) + K\left( {v\left( {x,f_{l} } \right)} \right),\quad l = 1,2, \ldots ,m.$$

If $R(x,f_{l} ) = 0$, the exact solution $v(x,f_{l} )$ is attained, currently if $R(x,f_{l} ) \ne 0,$ it can be minimized as follow

9 $$J(f_{l} ) = \int\limits_{c}^{d} {R^{2} (x,f_{l} )dx} .$$

where $c,\,d$ are constants, $f_{1} ,f_{2} ,f_{3} ,...$ can be attain as

10 $$\frac{\partial J}{{\partial f_{l} }} = 0,\quad l = 1,2,...,m.$$

After attaining these constants the approximate solution is attained from (7).

$f_{l}$ are fixed auxiliary parameters, Control the convergence region and rate of the homotopy series, they can be chosen optimally through residual minimization, they do not disturb the structure of the original problem, only the quality of approximation.

## Governing equations

The following are the basic incompressible couple stress fluid equations^24–26^

11 $$\nabla .V = 0,$$

12 $$\rho \mathop V\limits^{ \bullet } = \rho f - \nabla p - \nabla T,$$

13 $$\rho C_{p} \mathop \Theta \limits^{ \bullet } = k\nabla^{2} \Theta - T\,L,$$

Here $V$ is the velocity vector, $p$ is pressure, $f$ is the body force, fluid density is represented by $\rho$, $\kappa$ is conductivity, and $L,$ is the gradient of $V$ and the Material derivative is denoted by $\frac{D}{Dt}$,

14 $$\frac{D}{Dt}(\nu ) = \left( {\frac{\partial }{\partial t} + V.\nabla } \right)(\nu ).$$

The symbol ${\rm T}$ represents extra stress tensor as

15 $${\rm T} = \mu (A_{1} ) + \alpha_{1} (A_{2} ) + \alpha_{2} (A_{1}^{2} )$$

where $\alpha_{1} \,\& \,\,\alpha_{2}$ denotes the normal stress coefficients, and effective viscosity for second grade fluid is denoted by $\mu$ and can be defined as function of share stress as under

16 $$\mu = \eta \left( {\frac{1}{2}trA_{1}^{2} } \right)^{\frac{m}{2}}$$

where the flow consistency index is denoted by $\eta$ and $m$ is the flow behavior index, $A_{1} \& A_{2}$ are Rivlin Erickson tensors and given as

17 $$\begin{gathered} A_{1} = L + L^{t} , \hfill \\ A_{2} = \frac{{dA_{1} }}{dt} + A_{1} L + L^{t} A_{1} , \hfill \\ \end{gathered}$$

## Problem formulation

We have two cases in this paper, that is lifting and drainage case.

### Lifting case

**Fig. 1:**
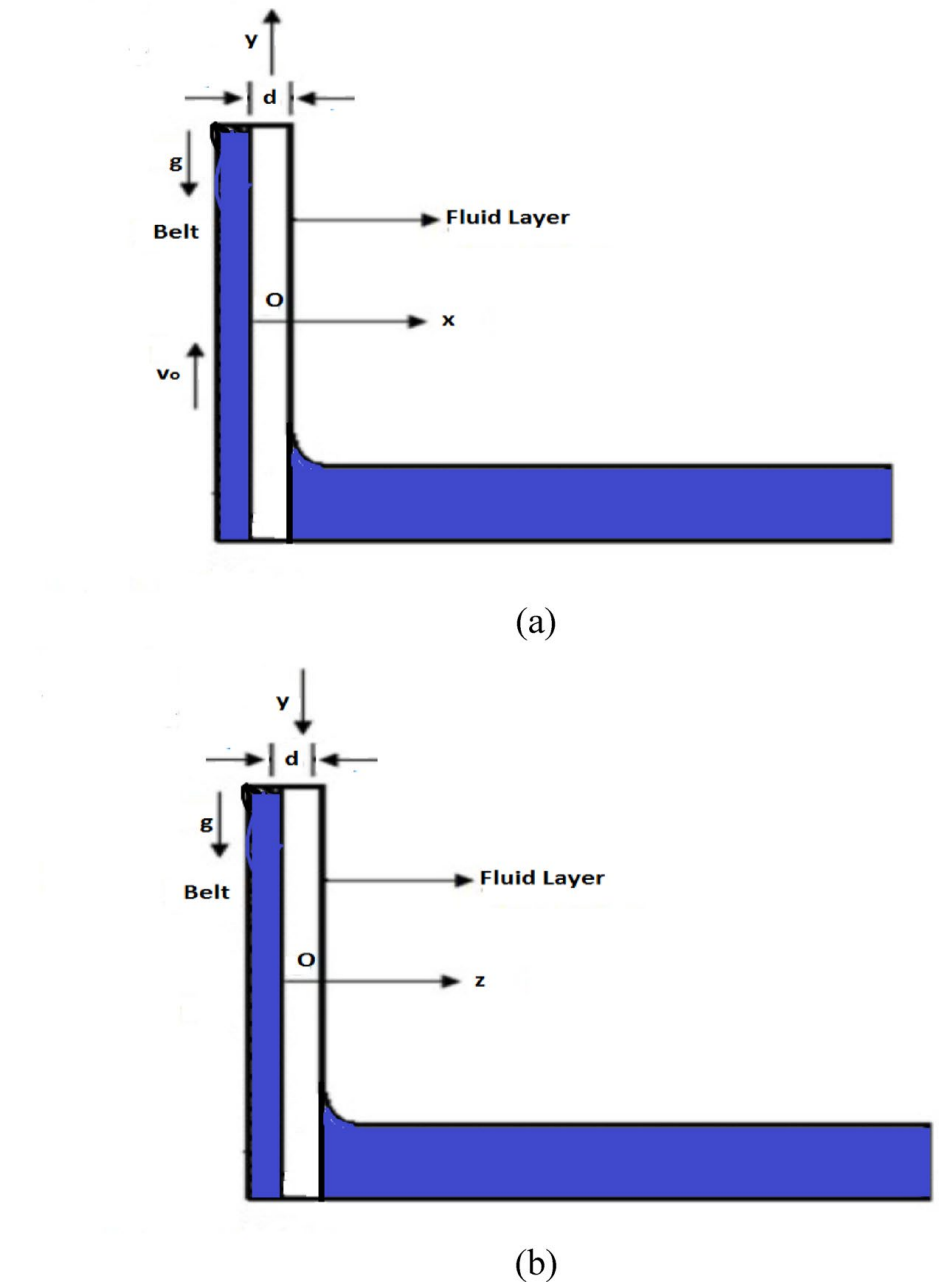
(**a**) Geometry of lifting problem. (**b**) Geometry of drainage problem.

Second-grade fluids denote a significant class of non-Newtonian fluids that account for normal stress differences and elastic effects absent in Newtonian models. These fluids are mostly applicable in describing the behavior of industrial lubricants, polymer melts, polymer solutions, and certain biological fluids, where non-Newtonian effects are noticeable and viscoelastic effects play a noteworthy role. Imagine a pot containing an incompressible, non-isothermal, modified second grade fluid. A belt moves upward at a constant speed $V_{0}$ passing through the container, as presented in Fig. 1a. While the belt is in motion, it transports with it a thin layer of the fluid, which has a uniform thickness $d.$ Gravity acts on this fluid layer, dragging it downward along the surface of the belt. To define the setup more specifically, we take a coordinate system where the x-axis is vertical to the belt surface, and the y-axis is along the direction of the belt. The origin (point O) is located on the belt surface at the level where the belt emerges from the fluid container, as presented in Fig. 1a. The flow is assumed to be uniform, laminar, steady, incompressible, and non-isothermal. The non-dimensional parameters governing the flow are the flow behavior index, $m$, Brinkman number $B_{r} .$ and Stokes number $s_{t} ,$ which describe the rheology, viscous heating, and inertial effects, respectively. Owing to the strong nonlinearity of the governing equations, the Asymptotic Homotopy Perturbation Method (AHPM) is employed to acquire approximate solutions for velocity and temperature profiles, along with for shear stress and flow rate. This formulation allows the efficient study of lifting thin film flow under realistic physical conditions. The velocity and temperature of the fluid in the thin layer are both described using specific profile functions.

18 $$V = (0,v(x),0),\,\,\Theta = \Theta (x).$$

Equations (11) is identically satisfied and Eqs. (2) implies

19 $$\frac{\partial p}{{\partial x}} = \left( {2\alpha_{1} + \alpha_{2} } \right)\frac{\partial }{\partial x}\left( {\frac{\partial v}{{\partial x}}} \right)^{2} ,$$

20 $$\frac{\partial p}{{\partial y}} = - \rho g + \eta \frac{\partial }{\partial x}\left( {\frac{\partial v}{{\partial x}}} \right)^{m + 1} ,$$

21 $$\frac{\partial p}{{\partial z}} = 0,$$

For the given problem the pressure $p$ is atmospheric pressure and is constant, thus $\frac{\partial p}{{\partial y}} = 0,$ so Eq. (20) becomes

22 $$\frac{d}{dx}\left( {\frac{dv}{{dx}}} \right)^{m + 1} = \frac{\rho g}{\eta },$$

Using Eqs. (18) in Eqs. (13) we have

23 $$\frac{{d^{2} \Theta }}{{dx^{2} }} + \frac{\eta }{k}\left( {\frac{dv}{{dx}}} \right)^{m + 2} = 0,$$

23a $$\begin{gathered} at\,\,x = 0\,\,v = V_{0} ,\,\Theta = \Theta_{0} , \hfill \\ at\,\,\,x = \delta ,\,\frac{dv}{{dy}} = 0,\frac{d\Theta }{{dx}} = 0. \hfill \\ \end{gathered}$$

The non-dimensional parameters

$$v^{*} = \frac{v}{V},\;x^{*} = \frac{x}{d},\;\Theta^{*} = \frac{{\Theta - \Theta_{0} }}{{\Theta_{1} - \Theta_{0} }},\;\mu^{*} = \frac{\mu }{{\mu_{0} }},\;S_{t} = \frac{{\rho gd^{2} }}{{V\mu_{0} }},\;B_{r} = \frac{{\mu_{0} V^{2} }}{{\kappa \left( {\Theta_{1} - \Theta_{0} } \right)}}.$$

where $S_{t} ,$ stoke number, and $\,B_{r}$ is brinkman number.

### Drainage problem

Now consider a situation where the fluid is flowing downward onto a vertical belt that is motionless, as presented in Fig. 1b. In this arrangement, we express the y-axis to run downward along the belt, and the z-axis to be vertical to the belt’s surface, with the origin positioned on the surface of the belt, as demonstrated in Fig. 1b. The fluid moves downward due to the force of gravity. In this case, the governing Eqs. (12) and (13) implies,

24 $$\frac{d}{dx}\left( {\frac{dv}{{dx}}} \right)^{m + 1} = - \frac{\rho g}{\eta },$$

25 $$\frac{{d^{2} \Theta }}{{dx^{2} }} + \frac{\eta }{k}\left( {\frac{dv}{{dx}}} \right)^{m + 2} = 0,$$

$$\begin{gathered} at\,\,z = 0\,\,v = 0,\,\Theta = \Theta_{0} , \hfill \\ at\,\,\,z = 1,\,\frac{dv}{{dz}} = 0,\frac{d\Theta }{{dz}} = 0, \hfill \\ \end{gathered}$$

## Solutions of the problem

The lifting velocity $({\mathrm{v}}_{L} )$ is of first order and lifting temperature $(\Theta_{L} )$ is of second order solutions employing AHPM are as under.

Zero order solution for lifting velocity using AHPM is

$$v_{L0} = v_{0}$$

First order solution for lifting velocity using AHPM is

$$v_{L1} = \left( {C_{1} \left( {1 + m} \right)s^{{\frac{1}{m + 1}}} } \right){{\left( {1 - \left( {1 - x} \right)^{{\frac{1}{m + 1}}} + \left( {\left( {1 - x} \right)^{{\frac{1}{m + 1}}} } \right)x} \right)} \mathord{\left/ {\vphantom {{\left( {1 - \left( {1 - x} \right)^{{\frac{1}{m + 1}}} + \left( {\left( {1 - x} \right)^{{\frac{1}{m + 1}}} } \right)x} \right)} {(2 + m)}}} \right. \kern-0pt} {(2 + m)}}.$$

$$v_{L} = v_{L0} + v_{L1} = v_{0} + \left( {C_{1} \left( {1 + m} \right)s^{{\frac{1}{m + 1}}} } \right){{\left( {1 - \left( {1 - x} \right)^{{\frac{1}{m + 1}}} + \left( {\left( {1 - x} \right)^{{\frac{1}{m + 1}}} } \right)x} \right)} \mathord{\left/ {\vphantom {{\left( {1 - \left( {1 - x} \right)^{{\frac{1}{m + 1}}} + \left( {\left( {1 - x} \right)^{{\frac{1}{m + 1}}} } \right)x} \right)} {\left( {2 + m} \right)}}} \right. \kern-0pt} {\left( {2 + m} \right)}}.$$

The value of $C_{1}$ is obtain using least square method, and using $m = 1,s = 1,V_{0} = 2$ in the above lifting velocity we have and in similar way lifting temperature is obtained.

26 $$v_{L} = 2 - 0.6666666666666666\left( {1 - \sqrt {1 - x} + (\sqrt {1 - x} )x} \right).$$

27 $$\begin{aligned} \Theta_{L} = & 2 + 1/70\left( { - 0.657267 + 0.657267\left( {1 - x} \right)^{3/2} } \right. \\ & \left. { - 1.31453\left( {1 - x} \right)^{3/2} x + 0.657267\left( {1 - x} \right)^{3/2} x^{2} } \right). \\ \end{aligned}$$

The Drainage velocity ${\mathbf{(}}v_{D} {\mathbf{)}}$ is of first order and Drainage temperature $(\Theta_{D} )$ is of second order solutions employing AHPM are as under

$$v_{D0} = 0,$$

First order solution for lifting velocity using AHPM is

$$v_{D1} = \left( {C_{1} (1 + m)s^{{\frac{1}{m + 1}}} } \right){{\left( {1 - (1 - x)^{{\frac{1}{m + 1}}} + \left( {(1 - x)^{{\frac{1}{m + 1}}} } \right)x} \right)} \mathord{\left/ {\vphantom {{\left( {1 - (1 - x)^{{\frac{1}{m + 1}}} + \left( {(1 - x)^{{\frac{1}{m + 1}}} } \right)x} \right)} {(2 + m)}}} \right. \kern-0pt} {(2 + m)}}.$$

$$v_{D} = v_{D0} + v_{D1} = \left( {C_{1} (1 + m)s^{{\frac{1}{m + 1}}} } \right){{\left( {1 - (1 - x)^{{\frac{1}{m + 1}}} + \left( {(1 - x)^{{\frac{1}{m + 1}}} } \right)x)} \right)} \mathord{\left/ {\vphantom {{\left( {1 - (1 - x)^{{\frac{1}{m + 1}}} + \left( {(1 - x)^{{\frac{1}{m + 1}}} } \right)x)} \right)} {(2 + m)}}} \right. \kern-0pt} {(2 + m)}}.$$

The value of $C_{1}$ is obtain using least square method and using $m = 1,s = 1,$ in the above drainage velocity we have and in similar way drainage temperature can be obtained.

28 $$v_{D} = 0.6666666666666666\left( {1 - \sqrt {1 - x} + (\sqrt {1 - x} )x} \right).$$

29 $$\begin{aligned} \Theta_{D} = & 2 + 1/70\left( {0.657267 - 0.657267(1 - x)^{3/2} } \right. \\ & \left. { + 1.31453(1 - x)^{3/2} x - 0.657267(1 - x)^{3/2} x^{2} } \right). \\ \end{aligned}$$

## Results and discussion

This paper examines the steady, incompressible, non-isothermal modified second grade fluid of the thin film for both lifting and drainage problem. Approximate solutions for both cases are attained, revealing that the solutions are dependent on the Brinkman number $B_{r}$, Stokes number $S_{t}$ and flow behavior index $m.$ The said parameters signify the shared effects of viscous dissipation, fluid inertia, and non-Newtonian material characteristics, respectively.

Table 1 shows the lifting velocity, lifting temperature distributions, and their corresponding residuals, whereas the drainage velocity, drainage temperature, and residuals are described for the drainage case. The small residuals validate the accuracy and convergence of the approximate solutions (Table 2). Tables 3 and 4 examine the influence of the Stokes number $S_{t}$ and flow behavior index $m,$ on lifting velocity. It is observed that increasing either parameter decreases the lifting velocity. In physical point of view, higher values of the Stokes number imply stronger inertial resistance comparative to viscous forces, which decreases upward motion in the lifting film. Correspondingly, an increase in the flow behavior index $m,$ boosts the effective viscosity of the modified second grade fluid, prominent to increased resistance against flow and hence a decrease in lifting velocity. Tables 5, 6 and 7 study the lifting temperature distributions for fluctuating Stokes number $S_{t}$ and flow behavior index $m,$ and Brinkman number $B_{r}$, the results show an inverse relationship between temperature and these parameters. This behavior can be endorsed to the decrease in fluid motion produced by higher inertia and viscosity, which deteriorates convective heat transport and decreases the temperature in the lifting film. Tables 8 and 9 examine the drainage velocity and determine that increasing the Stokes number $S_{t}$ and flow behavior index $m,$ and Brinkman number $B_{r}$ increases the drainage velocity. In the drainage formation, gravitational forces control the flow, and higher inertia and material strength support the downward motion of the fluid, subsequent in increased velocity. Likewise, Tables 10, 11 and 12 reveal that the drainage temperature rises with these parameters. This happens due to stronger fluid motion in the drainage case boosts viscous dissipation and convective heat transfer, prominent to higher temperature distributions. Tables 13 and 14 reveal the effectiveness of AHPM.

**Table 1:** The lifting velocity profile for $m = 1,s = 1,V_{0} = 2$, temperature distributions for $m = 1,B_{r} = 0.5,Q = 2,s = 0.3,V_{0} = 1$ and the associated residuals using AHPM.

$x$	AHPM $\left( {v_{L} } \right)$	Residual AHPM $({\mathrm{v}}_{L} )$	AHPM $(\Theta_{L} )$	Residual AHPM $(\Theta_{L} )$
0	2	0	2	1.38778 × 10^−17^
0.1	1.90254	0	1.9971	− 1.38778 × 10^−17^
0.2	1.81036	1.11022 × 10^−16^	1.99491	2.77556 × 10^−17^
0.3	1.72377	0	1.99331	0
0.4	1.64317	2.22045 × 10^−16^	1.99218	2.08167 × 10^−17^
0.5	1.56904	1.11022 × 10^−16^	1.99144	1.38778 × 10^−17^
0.6	1.50199	0	1.99099	− 3.46945 × 10^−18^
0.7	1.44288	1.11022 × 10^−16^	1.99075	1.73472 × 10^−18^
0.8	1.39296	0	1.99064	8.67362 × 10^−19^
0.9	1.35442	− 1.11022 × 10^−16^	1.99061	− 8.23994 × 10^−18^

**Table 2:** The Drainage velocity profile for $m = 1,s = 1,$, temperature distributions for $m = 1,B_{r} = 0.5,Q = 2,s = 0.3,$ and the associated residuals using AHPM.

$x$	AHPM $({\mathrm{v}}_{D} )$	AHPM $(\Theta_{D} )$	Residual AHPM $({\mathrm{v}}_{D} )$	Residual AHPM $(\Theta_{D} )$
0	0	2	0	− 1.38778 × 10^−17^
0.1	0.0974567	2.0029	0	1.38778 × 10^−17^
0.2	0.189639	2.00509	− 1.11022 × 10^−16^	− 2.77556 × 10^−17^
0.3	0.276225	2.00669	0	0
0.4	0.356828	2.00782	− 2.22045 × 10^−16^	− 2.08167 × 10^−17^
0.5	0.430964	2.00856	− 1.11022 × 10^−16^	− 1.38778 × 10^−17^
0.6	0.498012	2.00901	0	3.46945 × 10^−18^
0.7	0.557122	2.00925	− 1.11022 × 10^−16^	− 1.73472 × 10^−18^
0.8	0.607038	2.00936	0	− 8.67362 × 10^−19^
0.9	0.645585	2.00939	1.11022 × 10^−16^	8.23994 × 10^−18^

**Table 3:** Lifting velocity for different values of $s_{t}$ and $m = 0.1,\delta = 1,V_{0} = 1.$ using AHPM.

x	$s_{t} = 1$	$s_{t} = 2$	$s_{t} = 3$	$s_{t} = 4$
0.3	0.741316	0.514227	0.29771	0.0877856
0.4	0.673725	0.387301	0.114212	− 0.150563
0.5	0.61566	0.278263	− 0.0434269	− 0.355323
0.6	0.56728	0.187412	− 0.174771	− 0.525928
0.7	0.528786	0.115126	− 0.279277	− 0.661672
0.8	0.500444	0.061903	− 0.356222	− 0.761617
0.9	0.482648	0.0284849	− 0.404535	− 0.824371
1	0.47619	0.0163581	− 0.422067	− 0.847144

**Table 4:** Lifting velocity for different values of $m$ and $s_{t} = 0.1,\delta = 1,V_{0} = 1.$ using AHPM.

$x$	$m = 1$	$m = 2$	$m = 3$	$m = 4$
0.3	0.91265	0.868248	0.838173	0.81692
0.4	0.887161	0.82805	0.78769	0.759042
0.5	0.863717	0.790032	0.739275	0.703069
0.6	0.842515	0.754479	0.693235	0.649304
0.7	0.823823	0.721794	0.65001	0.598186
0.8	0.808038	0.692597	0.610297	0.55042
0.9	0.795848	0.668039	0.575425	0.507378
1	0.789181	0.651881	0.550127	0.474202

**Table 5:** Lifting temperature for different values of $s_{t}$ and $m = 1,\,B_{r} = 5,Q = 1$ using AHPM.

$x$	$s_{t} = 1$	$s_{t} = 2$	$s_{t} = 3$	$s_{t} = 4$
0.1	0.823766	0.0842602	− 0.970358	− 2.2639
0.2	0.690255	− 0.609481	− 2.46305	− 4.73655
0.3	0.592557	− 1.11714	− 3.55535	− 6.54595
0.4	0.524179	− 1.47244	− 4.31984	− 7.81233
0.5	0.479079	− 1.70678	− 4.82407	− 8.64759
0.6	0.451701	− 1.84904	− 5.13017	− 9.15464
0.7	0.437022	− 1.92532	− 5.29429	− 9.4265
0.8	0.430616	− 1.95861	− 5.36591	− 9.54514
0.9	0.428752	− 1.96829	− 5.38675	− 9.57966
1	0.428571	− 1.96923	− 5.38877	− 9.58301

**Table 6:** Lifting temperature for different values of $m$ and $s_{t} = 0.5,\,B_{r} = 0.3,Q = 1$ using AHPM.

$x$	$m = 1$	$m = 3$	$m = 5$	$m = 7$
0.1	0.996262	0.994999	0.994475	0.994189
0.2	0.993429	0.991103	0.990131	0.9896
0.3	0.991357	0.988163	0.986818	0.986081
0.4	0.989906	0.98603	0.984387	0.983484
0.5	0.98895	0.984564	0.982692	0.981661
0.6	0.988369	0.983629	0.981593	0.980469
0.7	0.988057	0.983096	0.980953	0.979768
0.8	0.987922	0.982843	0.980642	0.979422
0.9	0.987882	0.982761	0.980536	0.979302
1	0.987878	0.982751	0.980523	0.979286

**Table 7:** Lifting temperature for different values of $B_{r}$ and $s_{t} = 5,\,m = 1,Q = 1$ using AHPM.

$x$	$B_{r} = 1$	$B_{r} = 3$	$B_{r} = 5$	$B_{r} = 7$
0.1	0.605928	− 0.182215	− 0.970358	− 1.7585
0.2	0.30739	− 1.07783	− 2.46305	− 3.84827
0.3	0.0889293	− 1.73321	− 3.55535	− 5.37749
0.4	− 0.0639686	− 2.19191	− 4.31984	− 6.44778
0.5	− 0.164815	− 2.49444	− 4.82407	− 7.1537
0.6	− 0.226033	− 2.6781	− 5.13017	− 7.58223
0.7	− 0.258857	− 2.77657	− 5.29429	− 7.812
0.8	− 0.273182	− 2.81955	− 5.36591	− 7.91227
0.9	− 0.277349	− 2.83205	− 5.38675	− 7.94144
1	− 0.277753	− 2.83326	− 5.38877	− 7.94427

**Table 8:** Drainage velocity for different values of $s_{t}$ and $m = 0.1,\delta = 1,V_{0} = 1.$ using AHPM.

x	$s_{t} = 1$	$s_{t} = 2$	$s_{t} = 3$	$s_{t} = 4$
0.3	0.258684	0.485773	0.70229	0.912214
0.4	0.326275	0.612699	0.885788	1.15056
0.5	0.38434	0.721737	1.04343	1.35532
0.6	0.43272	0.812588	1.17477	1.52593
0.7	0.471214	0.884874	1.27928	1.66167
0.8	0.499556	0.938097	1.35622	1.76162
0.9	0.517352	0.971515	1.40453	1.82437
1	0.52381	0.983642	1.42207	1.84714

**Table 9:** Drainage velocity for different values of $m$ and $s_{t} = 0.1,\delta = 1,V_{0} = 1.$ using AHPM.

$x$	$m = 1$	$m = 2$	$m = 3$	$m = 4$
0.3	0.0873501	0.131752	0.161827	0.18308
0.4	0.112839	0.17195	0.21231	0.240958
0.5	0.136283	0.209968	0.260725	0.296931
0.6	0.157485	0.245521	0.306765	0.350696
0.7	0.176177	0.278206	0.34999	0.401814
0.8	0.191962	0.307403	0.389703	0.44958
0.9	0.204152	0.331961	0.424575	0.492622
1	0.210819	0.348119	0.449873	0.525798

**Table 10:** Drainage temperature for different values of $s_{t}$ and $m = 1,\,B_{r} = 5,Q = 1$ using AHPM.

$x$	$s_{t} = 1$	$s_{t} = 2$	$s_{t} = 3$	$s_{t} = 4$
0.1	1.17623	1.91574	2.97036	4.2639
0.2	1.30974	2.60948	4.46305	6.73655
0.3	1.40744	3.11714	5.55535	8.54595
0.4	1.47582	3.47244	6.31984	9.81233
0.5	1.52092	3.70678	6.82407	10.6476
0.6	1.5483	3.84904	7.13017	11.1546
0.7	1.56298	3.92532	7.29429	11.4265
0.8	1.56938	3.95861	7.36591	11.5451
0.9	1.57125	3.96829	7.38675	11.5797
1	1.57143	3.96923	7.38877	11.583

**Table 11:** Drainage Temperature for different values of $m$ and $s_{t} = 0.5,\,B_{r} = 0.3,Q = 1$ using AHPM.

$x$	$m = 1$	$m = 3$	$m = 5$	$m = 7$
0.1	1.00374	1.005	1.00553	1.00581
0.2	1.00657	1.0089	1.00987	1.0104
0.3	1.00864	1.01184	1.01318	1.01392
0.4	1.01009	1.01397	1.01561	1.01652
0.5	1.01105	1.01544	1.01731	1.01834
0.6	1.01163	1.01637	1.01841	1.01953
0.7	1.01194	1.0169	1.01905	1.02023
0.8	1.01208	1.01716	1.01936	1.02058
0.9	1.01212	1.01724	1.01946	1.0207
1	1.01212	1.01725	1.01948	1.02071

**Table 12:** Drainage Temperature for different values of $B_{r}$ and $s_{t} = 5,\,m = 1,Q = 1$ using AHPM.

$x$	$B_{r} = 1$	$B_{r} = 3$	$B_{r} = 5$	$B_{r} = 7$
0.1	1.39407	2.18222	2.97036	3.7585
0.2	1.69261	3.07783	4.46305	5.84827
0.3	1.91107	3.73321	5.55535	7.37749
0.4	2.06397	4.19191	6.31984	8.44778
0.5	2.16481	4.49444	6.82407	9.1537
0.6	2.22603	4.6781	7.13017	9.58223
0.7	2.25886	4.77657	7.29429	9.812
0.8	2.27318	4.81955	7.36591	9.91227
0.9	2.27735	4.83205	7.38675	9.94144
1	2.27775	4.83326	7.38877	9.94427

**Table 13:** The absolute error using exact solution and AHPM solution of Lifting velocity for $s_{t} = 1,m = 1,V_{0} = 2.$

x	AHPM $({\mathrm{v}}_{L} )$	Exact Sol $({\mathrm{v}}_{L} )$^27^	Error
0	2	2	0
0.1	1.90254	1.90254	4.16334 × 10^−17^
0.2	1.81036	1.81036	0
0.3	1.72377	1.72377	− 5.55112 × 10^−17^
0.4	1.64317	1.64317	0
0.5	1.56904	1.56904	0
0.6	1.50199	1.50199	0
0.7	1.44288	1.44288	0
0.8	1.39296	1.39296	0
0.9	1.35442	1.35442	0

**Table 14:** The absolute error using exact solution and AHPM solution of Drainage velocity for $s_{t} = 1,m = 1,V_{0} = 2.$

x	AHPM $({\mathrm{v}}_{D} )$	Exact Sol ${\mathrm{(v}}_{D} )$^27^	Error
0	0	0	0
0.1	0.0974567	0.0974567	− 4.16334 × 10^−17^
0.2	0.189639	0.189639	0
0.3	0.276225	0.276225	5.55112 × 10^−17^
0.4	0.356828	0.356828	0
0.5	0.430964	0.430964	0
0.6	0.498012	0.498012	0
0.7	0.557122	0.557122	0
0.8	0.607038	0.607038	0
0.9	0.645585	0.645585	0

**Fig. 2:**
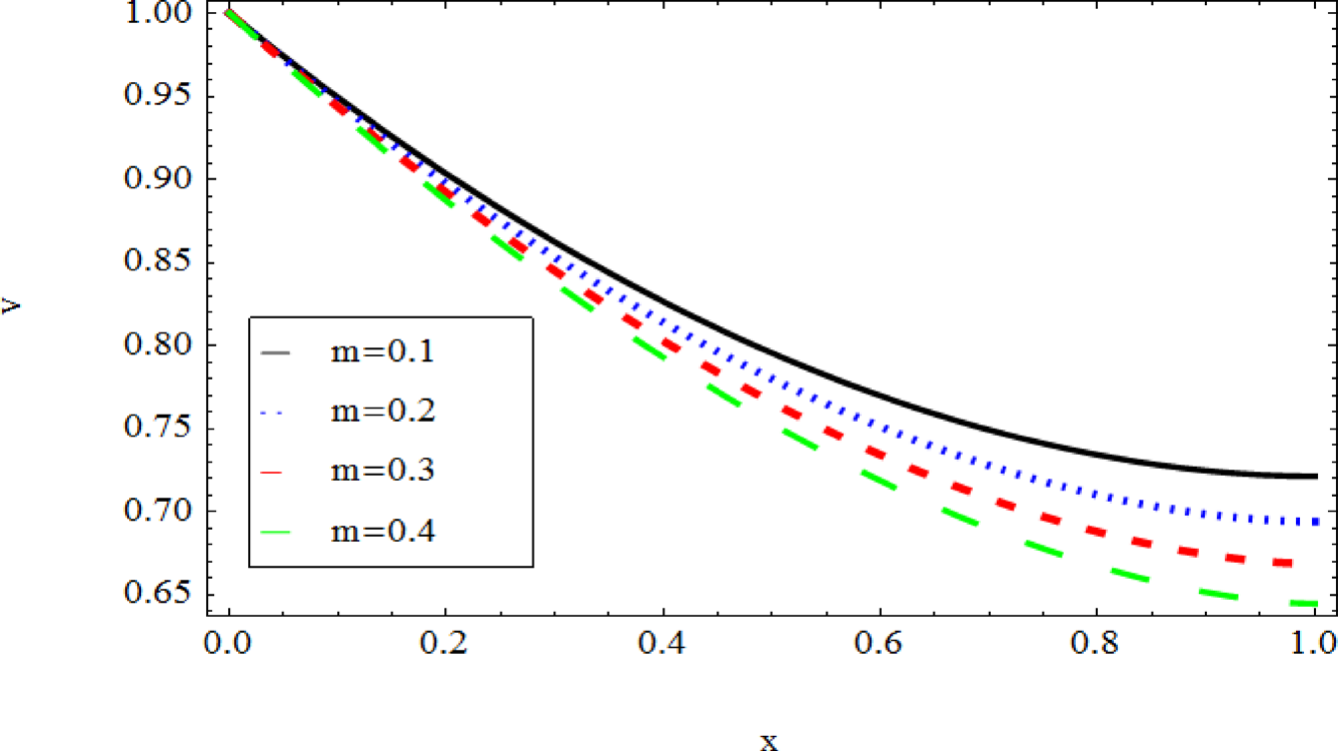
The Lifting Velocity for different values of $m$ and $s_{t} = 0.5,V_{0} = 1$ using AHPM.

**Fig. 3:**
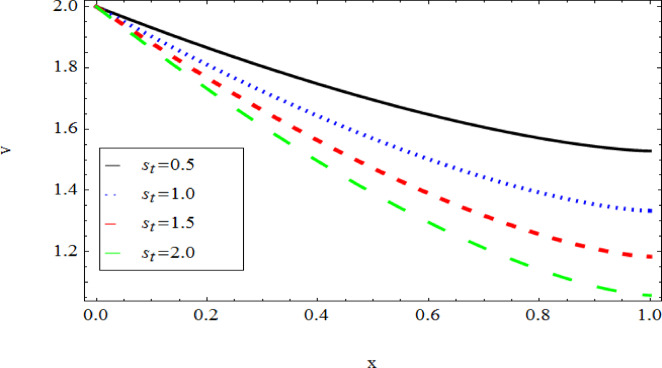
The Lifting Velocity for different values of $s_{t}$ and $m = 1,\delta = 1,V_{0} = 2$ using AHPM.

**Fig. 4:**
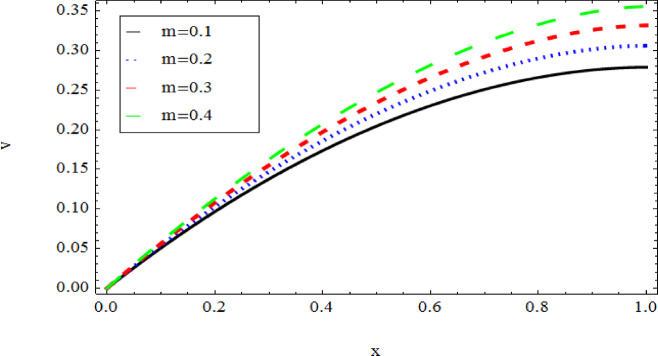
Drainage Velocity for different values of $m$ and $s_{t} = 0.5,$ using AHPM.

**Fig. 5:**
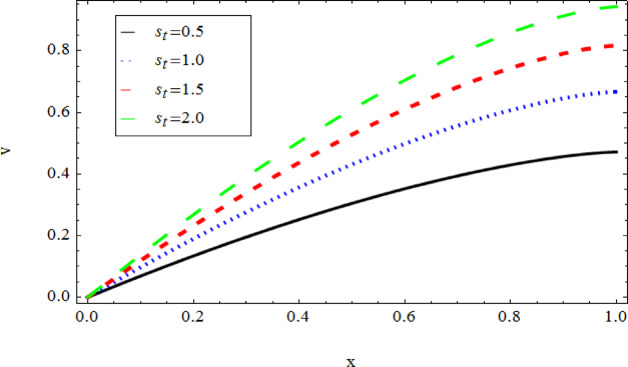
The Drainage Velocity for different values of $s_{t}$ and $m = 1,$ using AHPM.

**Fig. 6:**
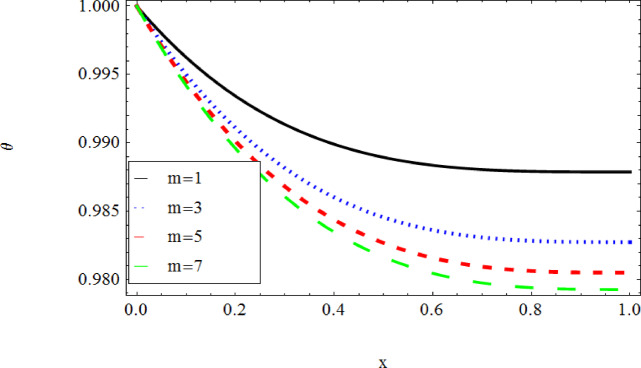
The Lifting Temperature for different values of $m$ and $s_{t} = 0.5,\,B_{r} = 0.3,Q = 1$ using AHPM.

**Fig. 7:**
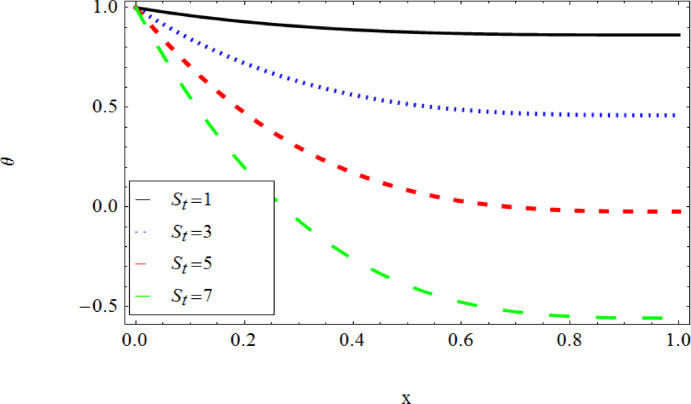
The Lifting Temperature for different values of $s_{t}$ and $m = 3,\,B_{r} = 1,Q = 1$ using AHPM.

**Fig. 8:**
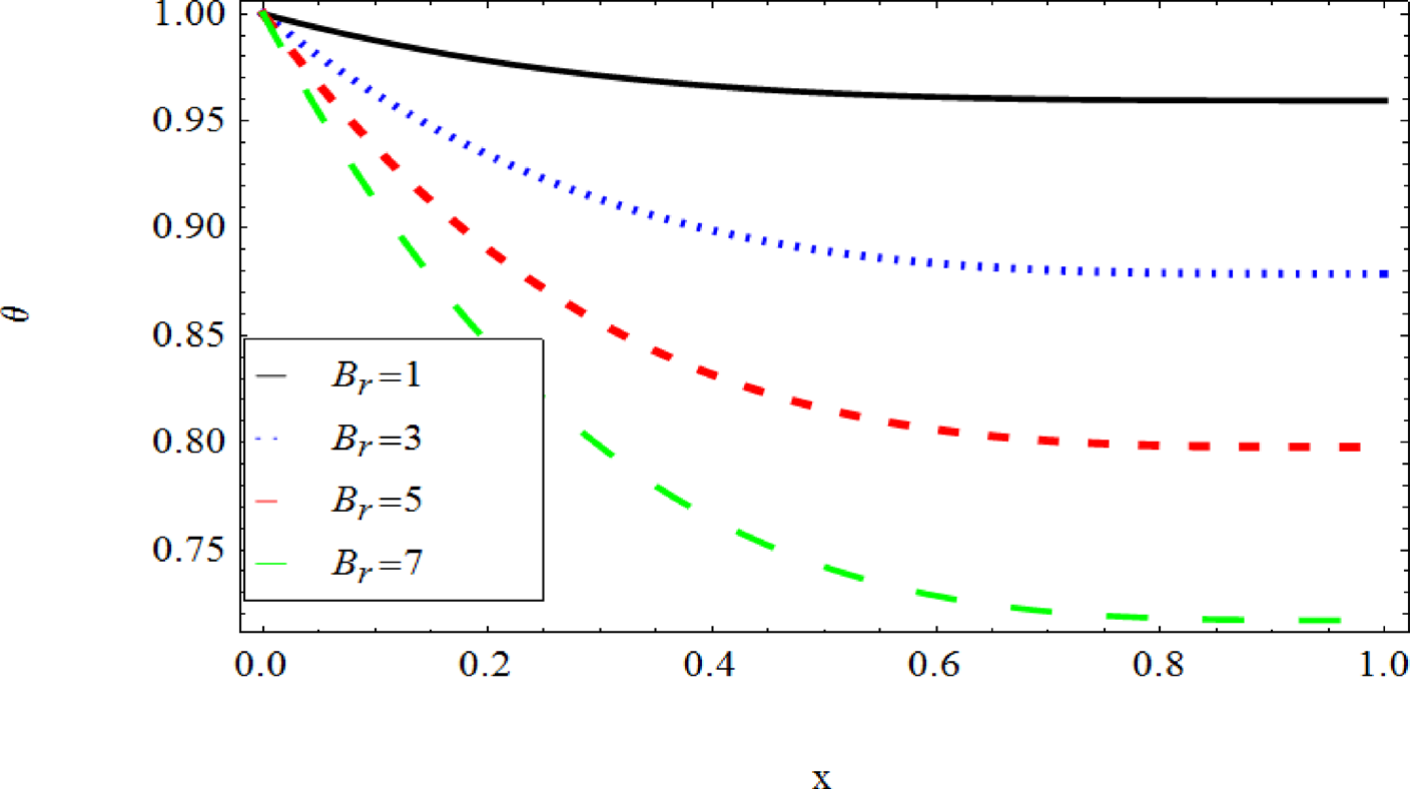
The Lifting Temperature for different values of $B_{r}$ and $m = 1,s_{t} = 0.5,Q = 1$ using AHPM.

**Fig. 9:**
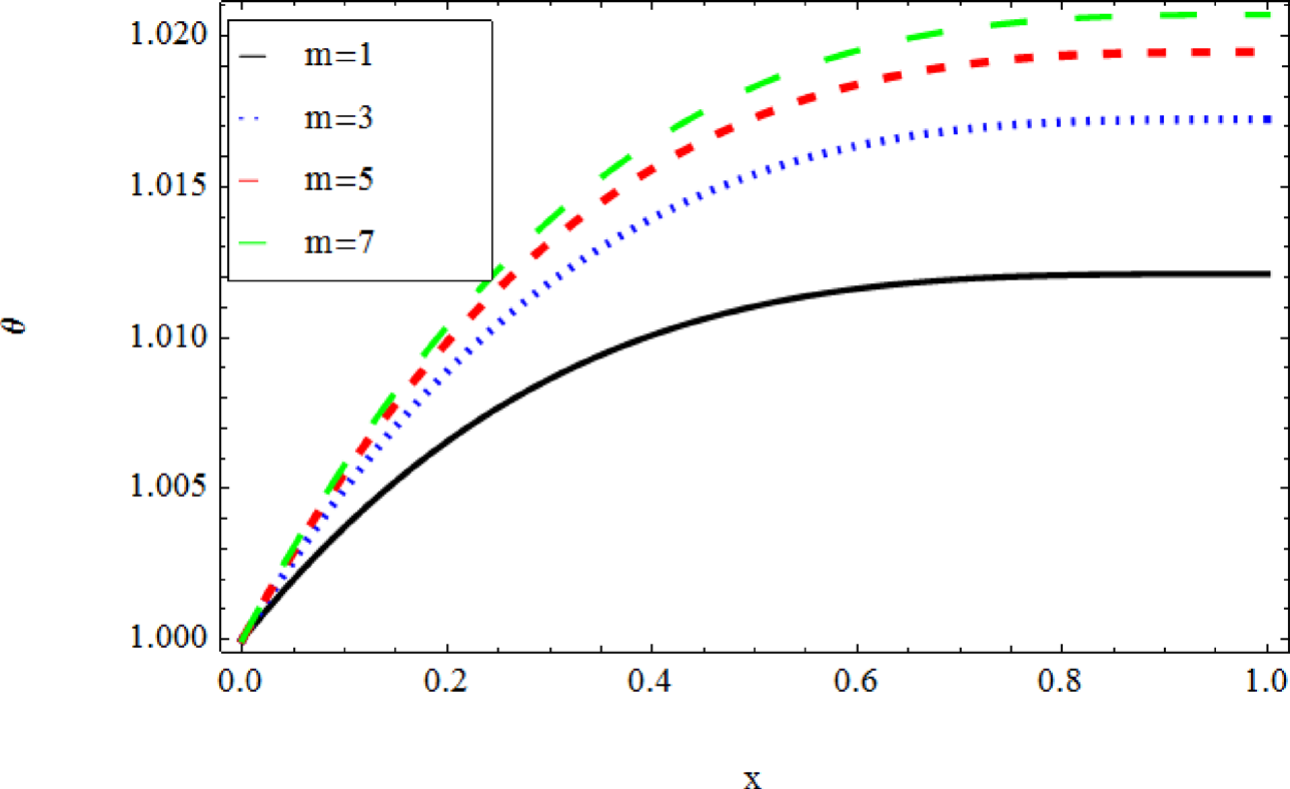
The Drainage Temperature for different values of $m$ and $s_{t} = 0.5,\,B_{r} = 0.3,Q = 1$ using AHPM.

**Fig. 10:**
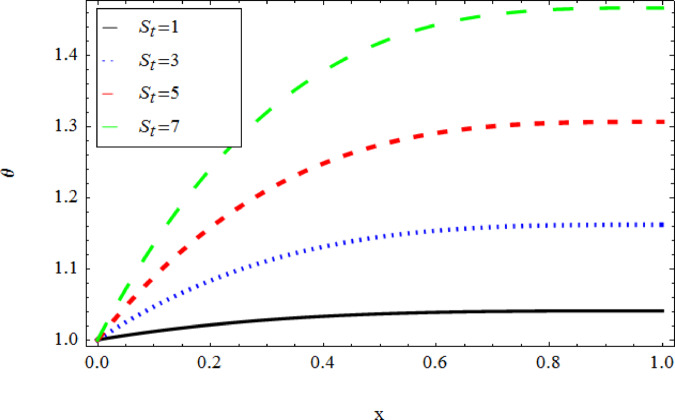
The Drainage Temperature for different values of $s_{t}$ and $m = 3,\,B_{r} = 1,Q = 1$ using AHPM.

**Fig. 11:**
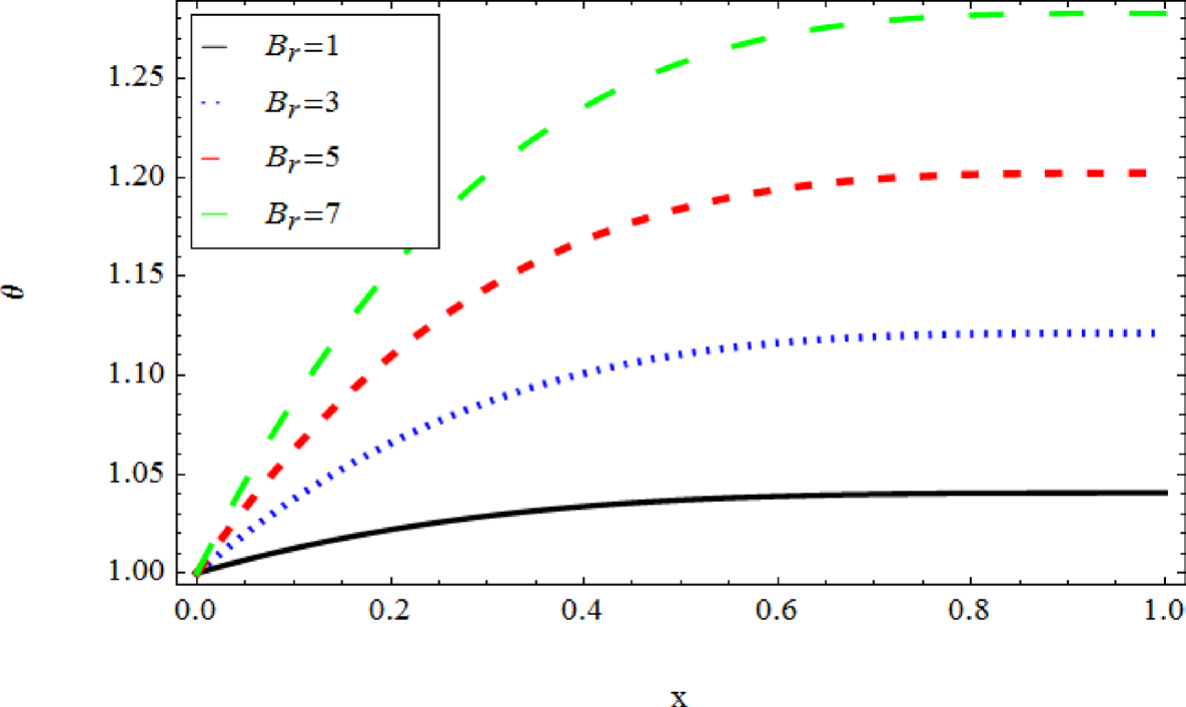
The Drainage Temperature for different values of $B_{r}$ and $m = 1,s_{t} = 0.5,Q = 1$ using AHPM.

**Fig. 12:**
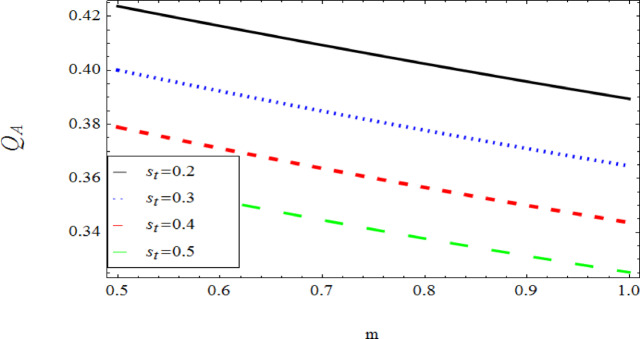
Lifting Flow rate for $s_{t}$ and ${\mathrm{m,}}$ using AHPM.

**Fig. 13:**
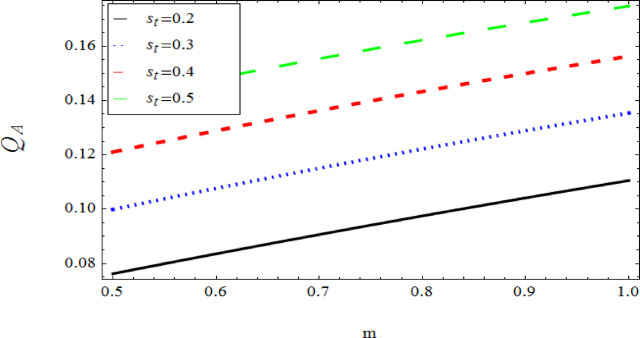
Drainage Flow rate for $s_{t}$ and ${\mathrm{m,}}$ using AHPM.

**Fig. 14:**
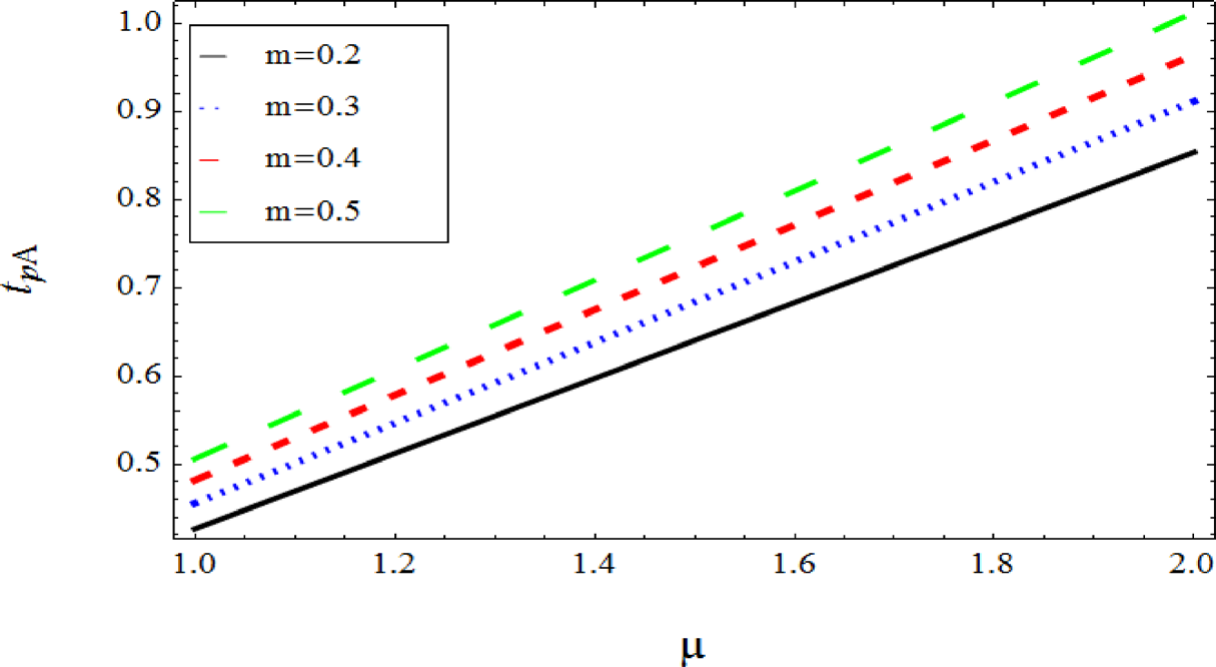
Lifting shear stress for parameters $m$ & $s_{t} = 0.4$ Using AHPM.

**Fig. 15:**
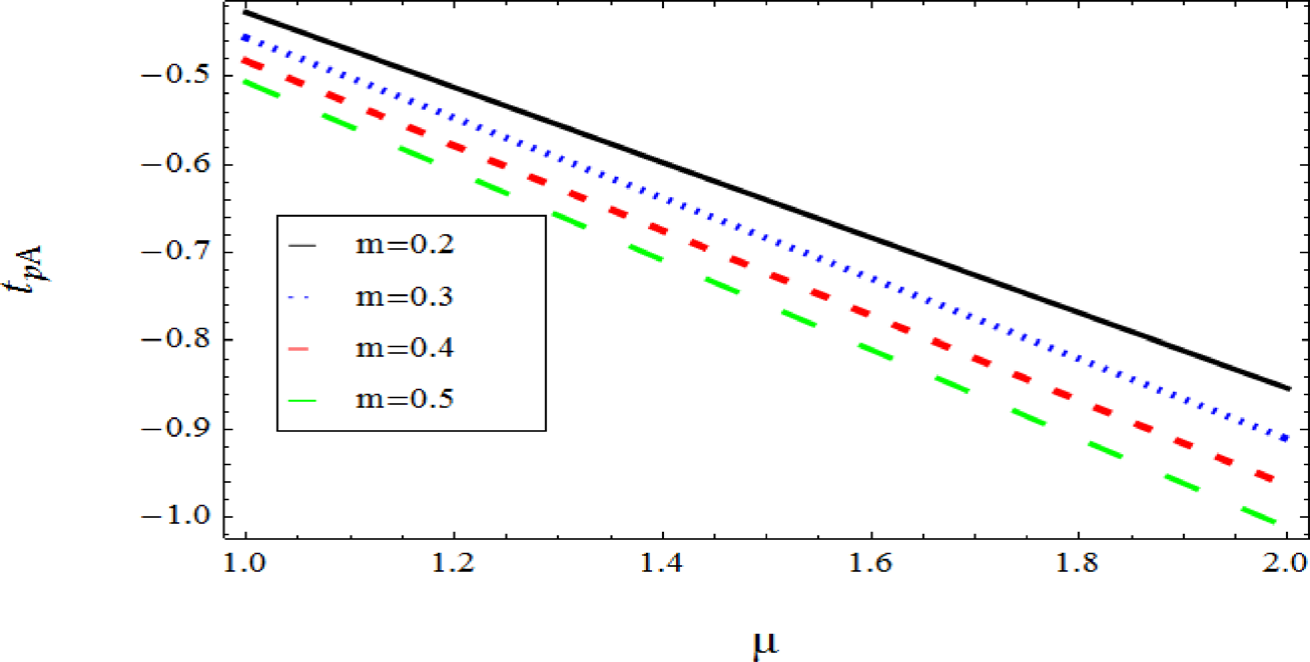
Drainage shear stress for parameters $m$ & $s_{t} = 0.4$ Using AHPM.

The graphical results further backing the numerical outcomes. Figures 2 and 3 display a decreasing tendency in lifting velocity with increasing flow behavior index $m,$ and Stokes number $S_{t}$, whereas Figs. 4 and 5 show the increasing of drainage velocity with the same parameter. Figures 6, 7 and 8 expose that lifting temperature decreases with increasing flow behavior index $m,$ and Stokes number $S_{t}$, endorsing the inverse relationship observed in the tables. Figures 9, 10, and 11 examine the behavior of parameters $B_{r} ,\,\,S_{t}$ and $m,$ on the drainage temperature and found that these parameters are directly related, it mean that drainage temperature increases with the said parameters due to enhanced downward flow and thermal energy generation. The flow rate for both lifting and drainage has been examined in Figs. 12 and 13 using parameter $S_{t}$ it is clear from these figures that $S_{t}$ is inversely related to flow rate in lifting case and directly related in drainage case. The shear stress has been investigated for lifting and drainage cases for parameter $m,$ and found that there is direct relation between parameter $m,$ and the lifting share stress, similarly an inverse relation is found with the drainage share stress as given in Figs. 14 and 15.

Generally, these results demonstrate the noteworthy role of material constants and non-Newtonian parameters in governing the velocity, temperature, flow rate, and shear stress characteristics of modified second grade fluid thin film flows.

## Conclusion

This paper explores the steady, incompressible and non-isothermal thin film flow of a modified second grade fluid under both conditions that is, lifting and drainage.. The governing highly non-linear ordinary differential equations with appropriate boundary conditions have been investigated and approximate solutions have been obtained for lifting velocity, temperature distributions, drainage velocity and drainage temperature distributions employing AHPM. Furthermore average velocity, shear stress and volume flow rate at the belt surface are presented graphically for both lifting and drainage configurations. At this point it is worth declaring that the flow of thin films are significantly influenced by $m$, $s_{t}$ and $B_{r} .$ The graphical and numerical analyses have been provided to explain the impact of various parameters on the fluid behavior. The effectiveness of AHPM is crystal clear from this research work.

The present work is appropriate to industrial processes concerning thin film transport of non-Newtonian fluids, for instance lubrication systems, coating flows, polymer processing, and thermal management uses where lifting and drainage mechanisms play a vital role.

*Future scope* The present study can be extended to unsteady and three-dimensional, with the effects of other physical parameters for example magnetic fields, thermal radiation, and slip boundary conditions may also be combined. Moreover, numerical and experimental studies can be made to confirm the approximate solutions.

## Data Availability

Data of this research and the code used in the analyses described in this paper are available from the corresponding author upon reasonable request.
